# Draft Genome Sequence of a Sulfate-Reducing Bacterium, “*Desulfofundulus salinum*” 435^T^, Isolated from a High-Temperature Gas Field in Russia

**DOI:** 10.1128/MRA.01408-18

**Published:** 2018-12-06

**Authors:** Denis S. Grouzdev, Salimat K. Bidzhieva, Tatiyana P. Tourova, Maria S. Krutkina, Andrey B. Poltaraus, Tamara N. Nazina

**Affiliations:** a Institute of Bioengineering, Research Center of Biotechnology, Russian Academy of Sciences, Moscow, Russian Federation; b Winogradsky Institute of Microbiology, Research Center of Biotechnology, Russian Academy of Sciences, Moscow, Russian Federation; c Engelhardt Institute of Molecular Biology, Russian Academy of Sciences, Moscow, Russian Federation; University of Maryland School of Medicine

## Abstract

The draft genome sequence of the thermophilic sulfate-reducing bacterium “Desulfofundulus salinum” strain 435^T^, isolated from condensate water of the Igrim high-temperature gas field (Western Siberia, Russia), is presented here. The genome is annotated to elucidate the taxonomic position of strain 435^T^.

## ANNOUNCEMENT

Gram-positive spore-forming sulfate-reducing bacteria of the genus Desulfotomaculum are commonly revealed in deep subsurface environments, including petroleum and gas reservoirs, by culture-based and molecular approaches (1, 2). Due to their ability to produce endospores and to grow on a range of organic substrates or H_2_-CO_2_ by reducing metals or sulfate and other oxidized sulfur compounds, or by fermentation, bacteria of the genus Desulfotomaculum (“D. salinum”, D. kuznetsovii, and D. thermocisternum) remain viable and geochemically active in deep anoxic environments (1, 3–6).

The thermophilic spore-forming sulfate-reducing Desulfotomaculum nigrificans strain 435^T^ was isolated in 1973 from gas condensate water of the Igrim gas field in Russia (4). Later, the halotolerant strain 435^T^ was reclassified as a new species, Desulfotomaculum salinum (7). Since the strain 435^T^ was deposited in a single international collection, this species is not validly described. On the phylogenetic tree of 16S rRNA gene sequences, strain 435^T^ forms an independent branch within the Desulfotomaculum genus cluster, sharing 94.9% similarity with the 16S rRNA gene of the most closely related type strain, Desulfotomaculum sp. strain DSM 6115^T^ (7–9). As a result of the recent revision of the genus Desulfotomaculum, species of the D. kuznetsovii cluster were assigned to the new genus Desulfofundulus (10). Strain 435^T^ is currently deposited in two microbial collections (VKM B 1492^T^ and DSM 23196). The aim of the present study was to sequence the genome of the strain 435^T^ in order to elucidate its taxonomic position.

Strain 435^T^ was grown anaerobically at 60°C in bicarbonate-buffered medium with lactate and sulfate as the substrates (7). Genomic DNA was extracted using Wilson’s method (11) with minor modifications. Cells were harvested from 2 liters of culture medium by centrifugation after 7 days of incubation, and the cell pellet was resuspended in 400 μl of Tris-EDTA (TE) buffer. Thereafter, 25 μl of 10% SDS and 20 μl of proteinase K solution were added, and the mixture was incubated at 37°C for 60 min. After incubation, 125 μl of 4 M NaCl, 160 μl of 5% cetyltrimethylammonium bromide (CTAB) and 20 μl of RNase (10 mg/ml) were added. The mixture was then incubated for 10 min at 65°C and cooled to room temperature; thereafter, the mixture was treated with chloroform, followed by centrifugation for 10 min at 9,000 × *g*. DNA from the supernatant was recovered by adding 0.6 volume of isopropanol. The dried DNA was dissolved in 50 μl of Milli-Q water (MQ). The libraries were constructed with the NEBNext DNA library prep reagent set for Illumina, according to the protocol for the kit. Sequencing was undertaken using the Illumina HiSeq 1500 platform with 250-bp single-end reads. Raw reads were quality checked with FastQC version 0.11.7 (12), and low-quality reads were trimmed using Trimmomatic version 0.36, with default settings (13). Subsequently, the quality-filtered reads were *de novo* assembled with SPAdes version 3.11.0 using the default settings (14). A total of 1,788,819 reads were assembled into 71 contigs larger than 500 bp. The MeDuSa scaffolder, with default parameters (15), was used to generate scaffolds from the contigs and to perform the mapping against Desulfofundulus kuznetsovii DSM 6115^T^ (GenBank assembly accession no. GCF_000214705) as a reference genome. The final assembled 2,886,683-bp-long genome comprised 10 scaffolds, with an *N*_50_ value of 2,856,498 bp, G+C content of 55.1 mol%, and coverage of 137×. Annotations of the contigs were carried out using the NCBI Prokaryotic Genome Annotation Pipeline (PGAP) (16), which identified 2,909 genes, 2,755 coding sequences, 101 pseudogenes, and 43 tRNA genes. The average nucleotide identity (ANI) (17) and digital DNA-DNA hybridization (dDDH) (18) values of 92.4% and 51.1%, respectively, to the genome of the closest strain D. kuznetsovii DSM 6115^T^ were below the criteria for assignment to separate species (95 to 96% for ANI and 70% for dDDH) (19), which indicate that strain 435^T^ belongs to a new Desulfofundulus species.

### Data availability.

This whole-genome shotgun project has been deposited at DDBJ/ENA/GenBank under the accession no. RBWE00000000. The version described in this paper is the first version, RBWE01000000. The raw FASTQ reads have been deposited in the NCBI SRA database under the accession no. SRR8069234.
